# Recurrent paratesticular giant liposarcoma: A case report and literature review

**DOI:** 10.3389/fsurg.2023.1171952

**Published:** 2023-04-27

**Authors:** Runmiao Hua, Liwei Zhao, Li Xu, Ji Sun, Jiaguo Huang, Qiyan Hua

**Affiliations:** ^1^ Department of Urology, Affiliated Xiaoshan Hospital, Hangzhou Normal University, Hangzhou, China; ^2^ Department of Urology, School of Medicine, Hangzhou Normal University, Hangzhou, China

**Keywords:** paratesticular liposarcoma, treatment, diagnosis, prognosis, case report

## Abstract

**Background:**

Primary paratesticular liposarcoma is rarely diagnosed among urinary tumors. In this study, through the retrospective analysis of clinical data and literature review, a case of recurrent paratesticular liposarcoma with lymph node metastasis after radical resection has been reported to explore novel strategies for the diagnosis, treatment and prognosis of this rare disease.

**Case summary:**

The present case involved a patient who was misdiagnosed as a left inguinal hernia for the first time two years ago, but was later diagnosed as mixed liposarcoma by using postoperative pathology. Currently, he is readmitted to the hospital with a recurrence of the left scrotal mass for more than 1 year. Combined with the patient's past medical history, we performed radical resection of the left inguinal and scrotal tumors and lymphadenectomy of left femoral vein. The postoperative pathology indicated that well-differentiated liposarcoma was accompanied by mucinous liposarcoma (about 20%), and lymph node metastasis of left femoral vein both of which occurred at the same time. After the operation, we recommended the patient to receive further radiation therapy, but the patient and his family refused, hence we followed up the patient closely for a long time. During the recent follow-up, the patient reported no complaints of discomfort, and no recurrence of mass in the left scrotum and groin area.

**Conclusion:**

After conducting extensive review of literature, we conclude that radical resection remains the key to treat primary paratesticular liposarcoma, while the significance of the lymph node metastasis is still unclear. The potential effects of postoperative adjuvant therapy depends on the pathological type, and hence close follow-up observation is essential.

## Introduction

Sarcomas account for approximately 15% of all the childhood malignancies, compared with 1% of all adult malignancies. The most common subtypes of soft tissue sarcoma (STS) include undifferentiated pleomorphic sarcoma (UPS), gastrointestinal stromal tumors (GIST), liposarcoma (LPS) and leiomyosarcoma (LMS). Liposarcoma is mainly composed of mesenchymal cells formed during the process of differentiation from adipoblast to adipocyte, ranking second among the soft tissue sarcomas, and accounting for about 15%–20% (1). In addition, with significantly different from lipoma in the subcutaneous fat layer, liposarcoma often occurs in the deep soft tissue, limbs and retroperitoneum. It mainly affects the middle-aged and elderly people and slightly more common in males than females (2, 3). Paratesticular liposarcoma (PLS) is a distinct kind of urinary liposarcoma, which is very rarely observed in the clinic. It mainly originates from the spermatic cord, testicular membrane or epididymis. Clinically, it usually presents itself as a painless soft tissue mass in the groin or scrotum, which is often misdiagnosed as inguinal hernia or testicular tumor (4–6). Moreover, due to the rarity and morphological heterogeneity of PLS, it is difficult to make a definite diagnosis before the surgery, while the local recurrence rate of PLS patients after inappropriate surgical resection is significantly high. According to the new National Comprehensive Cancer Network (NCCN) guidelines (2), LPS can be predominantly divided into four main subtypes based on the histomorphology of liposarcoma: atypical lipomatous tumor/well-differentiated liposarcoma (ALT/WDLPS), dedifferentiated liposarcoma (DDLPS), myxoid liposarcoma (MLPS) and pleomorphic liposarcoma(PLPS), and the degree of malignancy increases in turn.

At present, there is little clinical understanding about the clinical characteristics, diagnosis, treatment and prognosis of PLS. In this article, we report a case of recurrent left PLS in patient, who was misdiagnosed as indirect inguinal hernia entering the scrotum at the first presentation, but then relapsed and was admitted to the hospital 2 years after the surgery. In addition, combined with the related treatment experience and comprehensive literature review, we have attempted to clarify the clinical diagnosis and treatment of this rare disease.

## Case presentation

### Chief complaint

A 72-year-old Chinese man was admitted to our hospital again for more than one year because of the recurrence of the tumor in the left scrotum and groin area.

### Past medical history

3 years ago, the patient accidentally discovered a lump in his left groin area, which was about 4 cm × 5 cm in size at that time. No attention was paid to consult a doctor, and then it gradually enlarged and entered the scrotum, with a size of about 20 cm × 20 cm. The surface was not found to be swollen and tender, so it could not be absorbed into the abdominal cavity. 2 years ago, the patient first visited the general surgery department of our hospital. At that time, combined with B-mode ultrasound, he was diagnosed with indirect inguinal hernia, and agreed to undergo hernia repair for the patient. During the operation, there was no hernia sac bulge, but a solid tumor with the appearance of adipose tissue was found in the scrotum, most of which were soft, some of which were tough, yellow and wrapped around the testicles and epididymis. Therefore, we performed scrotal tumor resection for patients, completely removed the tumor base, spermatic cord as well as the testicular tissue, and performed high ligation of spermatic cord. It was extremely difficult to diagnose fast freezing pathology during operation. Postoperative routine pathology showed mixed liposarcoma, mainly WDLPS, accompanied by MLPS and DDLPS. The tumor size was 22 cm × 14 cm × 8 cm, and the blood vessels were rich in the tissues, which in the focal area showed tumor-like hyperplasia. The capsule was complete, and the surgical margin was observed to be negative. Immunohistochemistry revealed Ki-67(5%), P53(+), S-100(1+), CD68(−), CD34(−), SMA(−), PCNA(3+), CD56(−). Patients and their families refused further adjuvant treatment after the surgery. More than a year ago, the tumor in the left scrotum and groin area recurred. At present, he is admitted to the urology department of our hospital. Except the swelling of scrotum, he has no other major complaints.

### Past medical history

The patient has hypertension for more than three years and is taking hydrochlorothiazide once a day for the same. He is also suffering from tipe 2 diabetes mellitus for laat three years and is on acarbose 50 mg once a day and glimepiride 1 mg once a day. The patient did not have any other surgical history.

### Personal and family history

The patient did not have any addiction to tobacco and alcohol. And he has no genetic history of cancer.

### Physical examination

**Figure 1:**
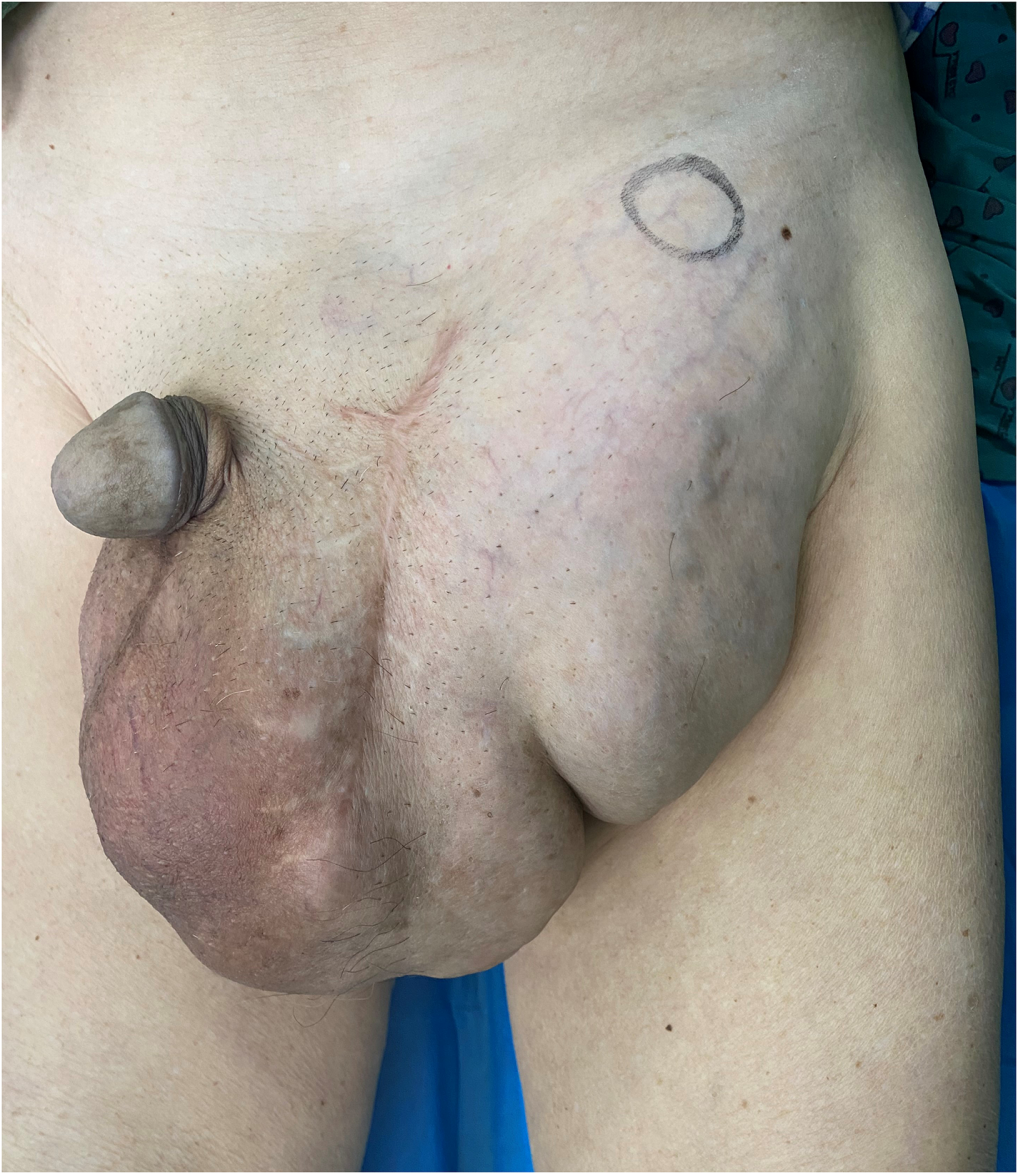
On physical examination, a lump of about 25 cm × 20 cm in size could be felt in the left scrotum and groin area, with tough texture and clear boundaries.

A lump of about 25 cm × 20 cm in size could be felt in the left scrotum and groin area, with tough texture and clear boundaries (Figure 1).

### Laboratory examination

After admission, the blood routine, coagulation function, liver as well as kidney function, tumor index and other blood biochemical tests of the patients showed no obvious abnormality.

### Imaging examination

**Figure 2:**
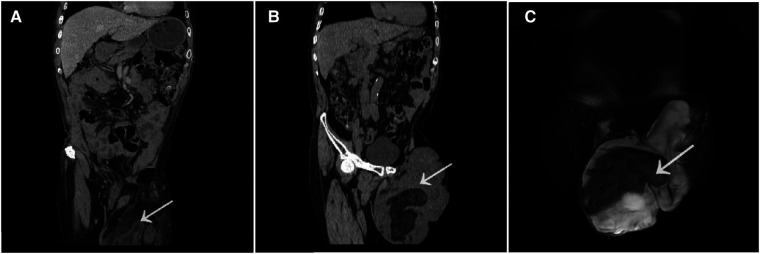
The abdominal enhancement CT showed that there was a lumpy uneven density shadow in the left scrotum, and there was fat and soft tissue density shadow In It which was not related with the contents of the abdominal cavity. After enhancement some nodules were slightly enhanced but the remaining enhancement was not found to be obvious [two years ago (**A**)]. The abdominal enhanced CT showed that there was a lumpy uneven density shadow in the left scrotum again,which was about 18.5 cm × 14 cm in size [now (**B**)]. MRI enhancement of the pelvic cavity showed irregular mass shadow in left groin and scrotum, lobulated, with some uneven fat signal in the center, about 20 cm × 13 cm × 17 cm in size. After enhancement, it was uneven and patchy, with arc-shaped enhancement,with no obvious changes in fat and cystic parts, clear edges, no connection between the mass and abdominal cavity (**C**). (The tumor tissue as indicated by arrowheads).

Two years ago, when the patient first visited a doctor in general surgery, abdominal enhancement computed tomography (CT) showed that there was a lumpy uneven density shadow in the left scrotum, and there was fat and soft tissue density shadow in it, which was not related with the contents of the abdominal cavity. After enhancement, some nodules were slightly enhanced, but the remaining enhancement was not found to be obvious. Soft tissue density nodules were found in the right groin area, but the enhancement was not obvious, and no enlarged lymph nodes were detected retroperitoneum (Figure 2A). After the recurrence, the patient visited our department again, and then the abdominal enhanced CT showed that there was a lumpy uneven density shadow in the left scrotum again, which was about 18.5 cm × 14 cm in size. The appearance was observed to be similar as observed during the first time, but a slightly larger lymph node was found in the left groin area (Figure 2B). Magnetic resonance imaging (MRI) enhancement of the pelvic cavity showed irregular mass shadow in left groin and scrotum, lobulated, with some uneven fat signal in the center, about 20 cm × 13 cm × 17 cm in size. After enhancement, it was uneven and patchy, with arc-shaped enhancement, with no obvious changes in fat and cystic parts, clear edges, no connection between the mass and abdominal cavity, slightly larger lymph nodes in both the groins, and uniform enhancement (Figure 2C).

### Final diagnosis

**Figure 3:**
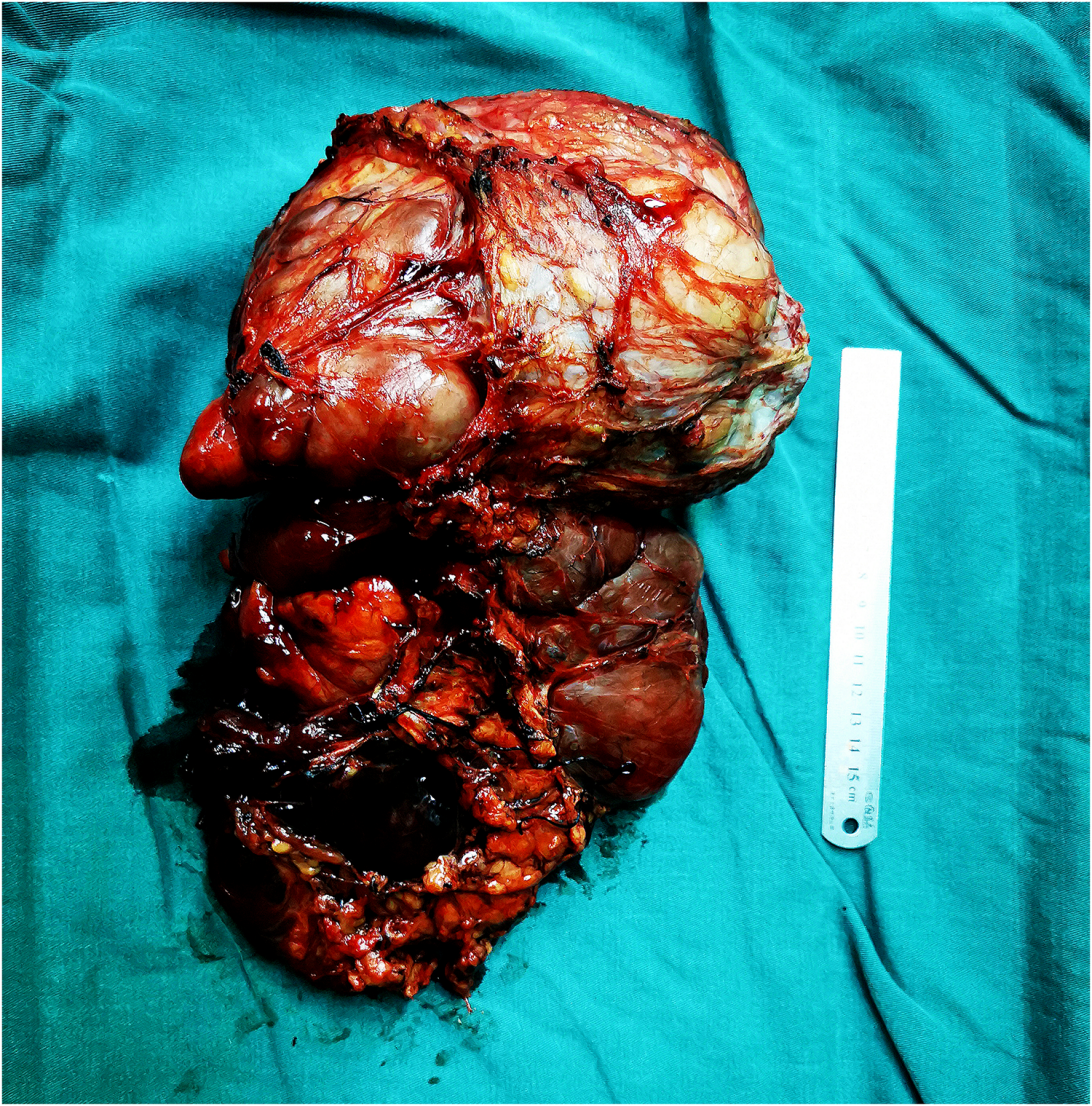
During the surgery, a tumor with a size of about 20 cm × 18 cm in the groin and scrotum was found, with a tough texture and a cystic mass, which was adhered to the surrounding tissues. After electrocoagulation with Ligsaure and separation, the tumor was completely removed.

**Figure 4:**
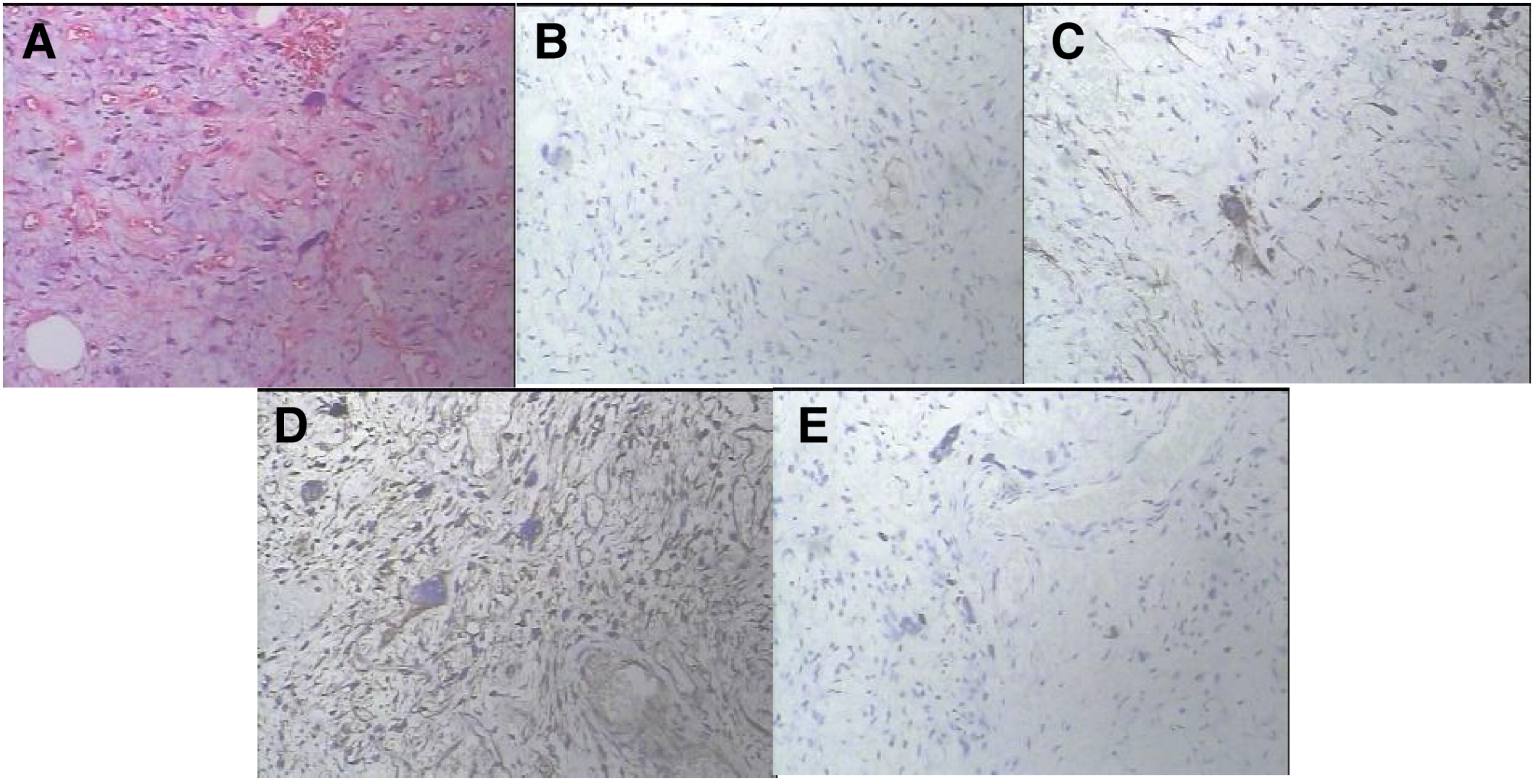
The histopathological findings and immunostaining. Postoperative routine pathology showed that left inguinal scrotal liposarcoma was primarily WDLPS, some of which were MLPS (about 20%), with a tumor size of 26 cm × 17 cm × 13 cm, accompanied by chronic inflammation of three lymph nodes around the tumor, and spindle cell malignant tumor of left femoral vein lymph nodes, which was considered as liposarcoma (**A**). On immunostaining, the tumor cells were focally positive for S-100 (**B**), Des (**C**), Vim (**D**) and Ki-67(5%) (**E**) and negative for CD34, Actin, CK, SMA, catenin-β and STAT6. (**A** × 100 magnifcation, **B–E** × 200 magnifcation).

Considering the recurrence of liposarcoma, radical tumor resection was performed. During the surgery, a tumor with a size of about 20 cm × 18 cm in the groin and scrotum was found, with a tough texture and a cystic mass, which was adhered to the surrounding tissues. After electrocoagulation with Ligsaure and separation, the tumor was completely removed (Figure 3). At the same time, we found that the lymph nodes along the left femoral vein were swollen and tough, so it was completely removed and sent for the pathological examination. About 50 ml of bleeding occurred during the operation, which lasted 2 h and 43 min, and the postoperative hospital stay was 12 days. Postoperative routine pathology showed that left inguinal scrotal liposarcoma was primarily WDLPS, some of which were MLPS (about 20%), with a tumor size of 26 cm × 17 cm × 13 cm, accompanied by chronic inflammation of three lymph nodes around the tumor, and spindle cell malignant tumor of left femoral vein lymph nodes, which was considered as liposarcoma (Figure 4A). Immunohistochemistry suggested CD34(−), S-100(scattered+) (Figure 4B), Des(partial+) (Figure 4C), Actin(−), CK(−), Vim(+) (Figure 4D), Ki-67(5%) (Figure 4E), P53(±), SMA(−), Myogenin(±), catenin-β(−), STAT6(−).

### Follow-up results

There were no related complications in the patient after the surgery. We recommended that the patient should undergo further radiotherapy, but the patient and his family still refused. Therefore, we could only follow up the patient closely. At present, up to more than one month before submission of the manuscript, the last reexamination of the patient showed that there was no recurrence of the lump in the scrotum and groin area, and CT scan exhibited no regional soft tissue density shadow. Further follow-up is in progress currently.

## Discussion

Sarcoma of genitourinary system is extremely rare malignancy, accounting for only 1%–2% of malignant tumors of genitourinary system. Most of them are diagnosed in children, and only a few cases are found in adults (7, 8). Among them, the most common tissue subtypes are leiomyosarcoma, liposarcoma and rhabdomyosarcoma, and the common sites of case reports are in the paratesticular area, kidney, prostate, penis and bladder (9). PLS is one of them, accounting for less than 12% of all liposarcoma. About 90% PLS have been reported to originate from the spermatic cord, followed by testis tunica vaginalis and epididymis (10, 11). In terms of the tumor volume, García Morúa et al. (12) reported that the tumor size could range from 1.5 cm to 23 cm, with the heaviest weight reaching 13 kg. This patient's lump had grown to the size of 22 cm × 14 cm × 8 cm at the initial onset, and recurred later to the size of 26 cm × 17 cm × 13 cm 2 years after the resection. For recurrent PLS, our case may be the largest described so far.

PLS usually presents itself as a slow-growing lump in the groin or scrotum, and the lump often grows progressively without pain and discomfort (13–16). Therefore, it is not easy for the patients to pay attention to it, leading to the symptoms being ignored and causing a delay in starting the treatment. Many patients exhibit a large tumor volume when seeing a doctor. The late symptoms are mainly related to the diameter as well as the location of the tumor and the invasion or compression of the surrounding organs (17). It is easy to be misdiagnosed as inguinal hernia or testicular or epididymal tumor during the first visit (12, 18). This patient, an elderly male, had no other special complaints except that of scrotal swelling caused by lump in inguinal area during the first visit. The surgeons and sonographers were preconceived, and the accuracy of specialist physical examination was not enough, which lead to the misdiagnosis of the tumor for a common left indirect inguinal hernia. Therefore, scrotal tumor was not found until the inguinal area was explored during the surgery. It was concluded that liposarcoma was common in middle-aged and elderly people, and its clinical manifestations were nonspecific, hidden and undetectable, which can be easily misdiagnosed.

Laboratory biochemical tests lack specificity in the diagnosis of PLS. Ultrasound is usually the first choice for imaging examination of the soft tissue tumors, and it plays a significant role in differentiating the cyst, hydrocele and epididymitis. PLS ultrasound usually shows solid, hyperechoic and heterogeneous lesions, but for the large tumors, identification of the origin and boundary of the tumor is often limited (19). Computed tomography scan and magnetic resonance imaging can aid to determine the complete location, histological and morphological features of the tumor as well to determine if the tumor has invaded adjacent tissues (20), and aid to distinguish the primary testicular tumors from the retroperitoneal lesions extending into the scrotum. MRI is currently the gold standard for staging the soft tissue tumors and is routinely employed to assess the extent of the local extension of the tumor (5). PLS on MRI T1WI and T2WI is usually dominated by the high signal, internal signal distribution is not uniform, visible low or isointense streak-like partition or small nodule shadow, and the signal of the lipid suppression sequence can be significantly reduced (18). The combination of T1-weighted and T2-weighted imaging, fat inhibition, tissue perfusion imaging, and water molecule diffusion-weighted imaging can effectively provide the anatomical structure, adjacent relationship, infiltration range, and information about the vascular as well as the nerve involvement of the tumor, and provide comprehensive and reliable information for the localization and qualitative diagnosis of the tumor (4, 21). Preoperative puncture is routinely recommended to confirm the pathological situation (22), especially for the tumors that cannot be removed after imaging evaluation, and biopsy to clarify the pathology can lead to the selection of other therapeutic approaches and methods. The diagnosis of PLS mainly depends on the postoperative histopathological, immunohistochemical and molecular tests. Pathology of the specimen shows that the tumor is generally large and lobulated, usually with a thin, discontinuous pseudocapsule, usually soft texture, section color, and necrosis depending on both the histological structure and pathological subtype. Microscopically, lipoblasts have been found to be vacuolated. MDM2 and CDK4 immunohistochemistry display a good application in the diagnosis of liposarcoma and accessory typing. BINH et al. (23) reported that the sensitivity and specificity of MDM2 gene amplification in the diagnosis of liposarcoma were 95% and 81%, respectively. The diagnostic sensitivity and specificity of CDK4 gene amplification were 92% and 95%, respectively. RICCIOTTI et al. (24) found that MDM2 and CDK4 gene amplification was associated with poor disease-specific and disease-free survival.

Histologically, the majority of liposarcoma cells can differentiate well and exhibit a low degree of malignancy. The results of two pathological examinations in our patient were mainly dominated by highly differentiated liposarcoma, with a partial manifestation of myxoid liposarcoma. The first pathology revealed a partially dedifferentiated liposarcoma, which was not observed after the recurrence, but with lymph node metastasis from the femoral vein. According to the prior reports in the literature, WDLPS is the most common subtype of LPS, accounting for about 40%–45% of LPS together with DDLPS (25). It is mostly found in limbs and retroperitoneum, but rarely can affect the in groin, mediastinum and head and neck. WDLPS is low in malignancy and generally has no metastatic potential, but the recurrence rate *in situ* after operation is significantly high (about 50%) (26) and some of them can gain metastatic ability after the process of dedifferentiation. Mucinous/round cell liposarcoma (MRCL) is the second most common subtype of LPS, accounting for about 30% of LPS. It is primarily composed of round cell liposarcoma and mucinous liposarcoma. The former is more invasive, so the higher the proportion of the round cells could be associated with the poor prognosis of MRCL patients. MRCL is common in thigh or popliteal fossa (75%), but rarely found in retroperitoneum. Its risk of distant metastasis is approximately 20%–40%, and it often can metastasizes to other soft tissues, abdominal cavity/retroperitoneal space and bone. MRCL is the most sensitive subtype of LPS to chemotherapy, and it is responsive to some extent in radiotherapy. Surgery combined with radiotherapy and chemotherapy can effectively control the growth of this subtype (27, 28). DDLPS accounts for 15%–20% of LPS. It is relatively a high-grade and highly invasive disease with high risk of local invasion, recurrence and metastasis. It is most commonly observed in retroperitoneum, followed by the limbs and spermatic cord/testicular area, but rarely seen in thoracic mediastinum and head and neck. About 90% of DDLPS are dedifferentiated at the onset, and 10% can be transformed from the recurrent WDLPS (29). The local recurrence rate is about 40%, and it has a metastasis rate of 20%–30% (30) and a mortality rate of 28%–30% (mainly due to the local recurrence). It can undergo metastasis to liver, lung, brain, bone (10%–15%), back, thigh and other soft tissue parts.

Thorough surgery to obtain the negative surgical margin is the common strategy for the treatment of histiocytoma at present. For PLS, the widely used surgical methods are radical orchiectomy with extensive local excision and high spermatic cord ligation (31). Incomplete excision can lead to the frequent tumor recurrence (32). After analyzing 265 PLS patients, Rei Kamitani et al. (33) showed that the recurrence-free survival rate of patients undergoing high inguinal resection was significantly higher in comparison to patients undergoing tumor resection. In addition, patients with positive margin under the microscope exhibited a markedly higher risk of recurrence than those with negative margin, and in the subgroup analysis of patients with positive margin, the potential effect of postoperative adjuvant radiotherapy on the recurrence-free survival rate was not found to be statistically significant. Coleman et al. (26) reported that nearly one-third of clinically asymptomatic patients with recurrence could be attributed to the tumor residues. Therefore, novel treatment options should extensively and thoroughly remove the surrounding tissues that may have infiltration, to effectively reduce the local recurrence rate and improve the tumor-free survival rate. Khandekar et al. (34) analyzed 25 patients with well-differentiated liposarcoma of spermatic cord (11 cases) and dedifferentiated liposarcoma (14 cases) and found that positive surgical margin was a bad prognostic factor for the local recurrence. They suggested that active reoperation should be taken for such patients. However, due to the scarcity of the cases, no common consensus has been reached on lymph node dissection, radiotherapy and chemotherapy (11, 35). It has been reported in the literature that PLS cases with lymph node metastasis are very rarely diagnosed. At present, it is believed that liposarcoma can spread and undergo metastasis mainly through infiltration of the spermatic cord and soft tissues around inguinal canal, or spread through blood metastasis, but rarely through lymph node metastasis (36, 37). Banowsky and Shultz et al. (38) reviewed 101 cases of spermatic sarcoma in order to evaluate the possible benefits of extensive lymphadenectomy, of which 29 cases showed local recurrence or were found to spread through blood. Among these patients, 17 patients had isolated lymphatic spread. However, lymph node dissection did not show any significant effect on the survival rate. Matei et al. (39) agreed that lymph node dissection has no significance impact in prolonging the recurrence interval and long-term survival. During the surgery of this patient, we found that the femoral vein lymph nodes were swollen, tough and abnormal, so we performed resection and sent the sample for pathological examination. Because the significance of lymph node dissection is unknown at present, we did not perform the lymph node dissection around the tumor.

At present, there is still controversy about the clinical effect of adjuvant radiotherapy or chemotherapy in PLS patients. Most scholars believe that preoperative or postoperative radiotherapy and chemotherapy are unable to substantially reduce the recurrence rate of WDLPS, and radiotherapy has no significance for localized lesions (39, 40), and is only applied to metastatic tumors or cases that could not be completely removed. However, it has been found that some types such as dedifferentiated liposarcoma and mucinous liposarcoma have better response to radiotherapy as compared to other subtypes of the tumors (31). Novel targeted therapies have also been used in the treatment of this rare disease. For example, a new cytotoxic drug, Ailibulin (41), targeted drug CDK4/6 inhibitor, MDM2 inhibitor, immune checkpoint inhibitor PD-1/PD-L1 (42) monoclonal antibody, etc. have been reported to show benefit in the treatment of dedifferentiated liposarcoma. Tribetidine, XPO1-1 inhibitor and NY-ESO-1 exhibited better curative effects on mucinous/round cell liposarcoma (43–45). In this case, the patient was elderly with severe underlying diseases, no metastasis was found in the auxiliary examination, and the family members refused chemotherapy and radiation, and selected to continue the regular follow-up.

The prognosis of patients mainly depends on the tumor site, tumor size, pathological type, classification and staging, and the degree of surgical resection. The tumor location is the most important factor for determining the prognosis. It has been reported in the literature that the tumor is primarily located in the retroperitoneum relative to the superficial parts such as limbs, and the risk of recurrence and metastasis is higher. PLS tumor diameter is an important factor for determining the success of radical surgical resection (46), especially when the tumor occurs in an anatomically constrained site. This is mainly because larger tumors can often compress or infiltrate the surrounding structures and tissues, thus making complete resection difficult, often leading to the positive surgical margins. Rodríguez et al. (13) used population-based large cancer registry to describe 168 cases of liposarcoma with good prognosis with disease-specific survival rates of 95% and 90% at 5 and 10 years, respectively. Multivariate analysis indicated that tumor grade, stage, histological type and lymph node metastasis served as independent predictors of prognosis. Rei Kamitani et al. (33) analyzed 265 PLS patients and suggested that the recurrence-free survival rate of patients who received high inguinal resection was significantly higher as compared to those patients who received tumor resection alone. In multivariate analysis, high inguinal resection significantly affected the recurrence-free survival rate (hazard ratio 0.16, *p* = 0.010), tumor size (hazard ratio 1.11, *p* = 0.017), dedifferentiation and round cell subtype (hazard ratio 7.56, *p* = 0.023 and hazard ratio 16.6, *p* = 0.021, respectively) were found to act as independent risk factors responsible for the tumor recurrence.

In summary, PLS is a rare tumor, which predominantly presents itself as irregular masses with different sizes and medium-soft texture in the scrotum, spermatic cord or groin in clinical. The laboratory tests are mostly negative, as well as heterogeneous and solid lesions can be observed based on imaging examinations. It can be easily misdiagnosed before the surgery, and careful physical examination is necessary. The possibility of PLS should also be considered in the identification of the fat-containing inguinal scrotal masses. The imaging procedures such as Ultrasound, CT and MRI can provide relevant imaging information on the nature of pre-operative liposarcoma. Immunohistochemistry and molecular testing of MDM2 and CDK4 can contribute to an accurate diagnosis. Therefore, based on the literature review, we suggest that in the case of rapid intraoperative cryosurgery with a definite pathology, extensive local excision and radical orchiectomy with high ligation of the spermatic cord in order to obtain a negative margin should be conducted as the best therapeutic strategy to maximize the chance of surgical cure. Incomplete resection can result in high local recurrence rate and shortened recurrence time after PLS resection. The histopathological type and malignant grade could be helpful to judge the prognosis, and further adjuvant treatment should be selected according to the different pathological types. Novel targeted therapies may be promising, but the long-term close patient follow-up is essential for optimal treatment.

## Data Availability

The raw data supporting the conclusions of this article will be made available by the authors, without undue reservation.

## References

[1] Ducimetière F Lurkin A Ranchère-Vince D Decouvelaere AV Péoc'h M Istier L Incidence of sarcoma histotypes and molecular subtypes in a prospective epidemiological study with central pathology review and molecular testing. PLoS One. (2011) 6(8):e20294. 10.1371/journal.pone.0020294 21826194 PMC3149593

[2] von Mehren M Randall RL Benjamin RS Boles S Bui MM Conrad EU 3rd Soft tissue sarcoma, version 2.2016, NCCN clinical practice guidelines in oncology. J Natl Compr Canc Netw. (2016) 14(6):758–86. 10.6004/jnccn.2016.0078 27283169

[3] Zhao C Han Z Xiao H Yang C Zhao Y Fan T Surgical management of spinal liposarcoma: a case series of 7 patients and literature review. Eur Spine J. (2016) 25(12):4088–93. 10.1007/s00586-015-4374-3 26781842

[4] Vukmirović F Zejnilović N Ivović J. Liposarcoma of the paratesticular tissue and spermatic cord: a case report. Vojnosanit Pregl. (2013) 70:693–6. 10.2298/VSP1307695V 23984620

[5] Schoonjans C Servaes D Bronckaers M. Liposarcoma scroti: a rare paratesticular tumor. Acta Chir Belg. (2016) 116:122–5. 10.1080/00015458.2016.1139939 27385300

[6] Gabriele R Ferrara G Tarallo MR Giordano A De Gori A Izzo L Recurrence of paratesticular liposarcoma: a case report and review of the literature. World J Surg Oncol. (2014) 12:276. 10.1186/1477-7819-12-276 25175606 PMC4168198

[7] Global Burden of Disease Cancer Collaboration, Fitzmaurice C Dicker D Pain A Hamavid H Moradi-Lakeh M MacIntyre MF The global burden of cancer 2013. JAMA Oncol. (2015) 1:505–27. 10.1001/jamaoncol.2015.0735 26181261 PMC4500822

[8] Dotan ZA Tal R Golijanin D Snyder ME Antonescu C Brennan MF Russo P. Adult genitourinary sarcoma: the 25-year memorial sloan-kettering experience. J Urol. (2006) 176:2033–8; discussion 2038–9. 10.1016/j.juro.2006.07.021 17070247

[9] Mondaini N Palli D Saieva C Nesi G Franchi A Ponchietti R Clinical characteristics and overall survival in genitourinary sarcomas treated with curative intent: a multicenter study. Eur Urol. (2005) 47(4):468–73. 10.1016/j.eururo.2004.09.013 15774243

[10] Bhangoo MS Cheng B Botta GP Thorson P Kosty MP. Reversible intrahepatic cholestasis in metastatic prostate cancer: an uncommon paraneoplastic syndrome. Mol Clin Oncol. (2018) 8(4):609–12. 10.3892/mco.2018.1564 29541472 PMC5838296

[11] Gatto L Del Gaudio M Ravaioli M Cescon M Tonini V Cervellera M Paratesticular mesenchymal malignancies: a single-center case series, clinical management, and review of literature. Integr Cancer Ther. (2020) 19:1534735419900554. 10.1177/1534735419900554 32009477 PMC7050957

[12] García Morúa A Lozano Salinas JF Valdés Sepúlveda F Zapata H Gómez Guerra LS. Liposarcoma of the espermatic cord: our experience and review of the literature. Actas Urol Esp. (2009) 33:811–5. 10.1016/S0210-4806(09)74235-4 19757668

[13] Rodríguez D Barrisford GW Sanchez A Preston MA Kreydin EI Olumi AF. Primary spermatic cord tumors: disease characteristics, prognostic factors, and treatment outcomes. Urol Oncol. (2014) 32(1):52.e19–25. 10.1016/j.urolonc.2013.08.009 24239475

[14] Goldberg H Wong LM Dickson B Catton C Yap SA Alkasab T Long-term oncological outcomes of patients with paratesticular sarcoma. BJU Int. (2019) 124:801–10. 10.1111/bju.14775 31001920

[15] Montgomery E Fisher C. Paratesticular liposarcoma: a clinicopathologic study. Am J Surg Pathol. (2003) 27(1):40–7. 10.1097/00000478-200301000-00005 12502926

[16] Li Z Zhou L Zhao L Chen P Liu Y Ding Y Giant paratesticular liposarcoma: a case report and review of the literature. Mol Clin Oncol. (2018) 8(4):613–6. 10.3892/mco.2018.1577 29556392 PMC5844082

[17] Khoubehi B Mishra V Ali M Motiwala H Karim O. Adult paratesticular tumours. BJU Int. (2002) 90(7):707–15. 10.1046/j.1464-410x.2002.02992.x 12410753

[18] Fitzgerald S Maclennan GT. Paratesticular liposarcoma. J Urol. (2009) 181(1):331–2. 10.1016/j.juro.2008.10.080 19010493

[19] De Nardi P Bissolati M Cristallo M Staudacher C. Recurrent giant liposarcoma of the spermatic cord. Urology. (2012) 79(1):113–4. 10.1016/j.urology.2011.02.004 21492916

[20] Woodward PJ Schwab CM Sesterhenn IA. From the archives of the AFIP: extratesticular scrotal masses: radiologic-pathologic correlation. Radiographics. (2003) 23(1):215–40. 10.1148/rg.231025133 12533657

[21] Pergel A Yucel AF Aydin I Sahin DA Gucer H Kocakusak A. Paratesticular liposarcoma: a radiologic pathologic correlation. J Clin Imaging Sci. (2011) 1:57. 10.4103/2156-7514.90952 22267992 PMC3261607

[22] Ikoma N Torres KE Somaiah N Accuracy of preoperative percutaneous biopsy for the diagnosis of retroperitoneal liposarcoma subtypes. Ann Surg Oncol. (2015) 22(4):1068–72. 10.1245/s10434-014-4210-8 25354575 PMC4520392

[23] Ikoma N Torres KE Somaiah N Hunt KK Cormier JN Tseng W MDM2 And CDK4 immunostainings are useful adjuncts in diagnosing well-differentiated and dedifferentiated liposarcoma subtypes: a comparative analysis of 559 soft tissue neoplasms with genetic data. Am J Surg Pathol. (2005) 29(10):1340–7. 10.1097/01.pas.0000170343.09562.39 16160477

[24] Binh MB Sastre-Garau X Guillou L de Pinieux G Terrier P Lagacé R High amplification levels of MDM2 and CDK4 correlate with poor outcome in patients with dedifferentiated liposarcoma: a cytogenomic microarray analysis of 47 cases. Cancer Genet. (2017) 218–219:69–80. 10.1016/j.cancergen.2017.09.005 29153098

[25] Ricciotti RW Baraff AJ Jour G Kyriss M Wu Y Liu Y Sarcoma classification: an update based on the 2013 world health organization classification of tumors of soft tissue and bone. Cancer. (2014) 120(12):1763–74. 10.1002/cncr.28657 24648013

[26] Coleman J Brennan MF Alektiar K Russo P. Adult spermatic cord sarcomas: management and results. Ann Surg Oncol. (2003) 10(6):669–75. 10.1245/aso.2003.11.014 12839852

[27] Di Giandomenico S Frapolli R Bello E Mode of action of trabectedin in myxoid liposarcomas. Oncogene. (2014) 33(44):5201–10. 10.1038/onc.2013.462 24213580

[28] Di Giandomenico S Frapolli R Bello E Uboldi S Licandro SA Marchini S Excellent local control rates and distinctive patterns of failure in myxoid liposarcoma treated with conservation surgery and radiotherapy. Int J Radiat Oncol Biol Phys. (2008) 70(3):760–5. 10.1016/j.ijrobp.2007.07.2337 17892916

[29] Guadagnolo BA Zagars GK Ballo MT Patel SR Lewis VO Benjamin RS Dedifferentiated liposarcoma: updates on morphology, genetics, and therapeutic strategies. Adv Anat Pathol. (2016) 23(1):30–40. 10.1097/PAP.0000000000000101 26645460

[30] Lee ATJ Thway K Huang PH Jones RL. Clinical and molecular Spectrum of liposarcoma. J Clin Oncol. (2018) 36(2):151–9. 10.1200/JCO.2017.74.9598 29220294 PMC5759315

[31] Schwartz SL Swierzewski SJ 3rd Sondak VK Grossman HB. Liposarcoma of the spermatic cord: report of 6 cases and review of the literature. J Urol. (1995) 153(1):154–7. 10.1097/00005392-199501000-00055 7966755

[32] Li F Tian R Yin C Liposarcoma of the spermatic cord mimicking a left inguinal hernia: a case report and literature review. World J Surg Oncol. (2013) 11:18. 10.1186/1477-7819-11-18 23351168 PMC3585812

[33] Li F Tian R Yin C Dai X Wang H Xu N Guo K. Optimal treatment strategy for paratesticular liposarcoma: retrospective analysis of 265 reported cases. Int J Clin Oncol. (2020) 25(12):2099–106. 10.1007/s10147-020-01753-3 32715355

[34] Khandekar MJ Raut CP Hornick JL Wang Q Alexander BM Baldini EH. Paratesticular liposarcoma: unusual patterns of recurrence and importance of margins. Ann Surg Oncol. (2013) 20(7):2148–55. 10.1245/s10434-013-2963-0 23591942

[35] Keenan RA Nic An Riogh AU Stroiescu A Paratesticular sarcomas: a case series and literature review. Ther Adv Urol. (2019) 11:1756287218818029. 10.1177/1756287218818029 30671140 PMC6329018

[36] Keenan RA Nic An Riogh AU Stroiescu A Fuentes A Heneghan J Cullen IM Paratesticular liposarcoma: a case report and review of the literature. Case Rep Urol. (2013) 2013:806289. 10.1155/2013/806289 23533935 PMC3600269

[37] Casali PG Abecassis N Aro HT Soft tissue and visceral sarcomas: eSMO-EURACAN clinical practice guidelines for diagnosis, treatment and follow-up [published correction appears in Ann Oncol. 2018 Oct 1;29(Suppl 4):iv268–iv269]. Ann Oncol. (2018) 29(Suppl 4):iv51–67. 10.1093/annonc/mdy096 30285214

[38] Casali PG Abecassis N Aro HT Bauer S Biagini R Bielack S Sarcoma of the spermatic cord and tunics: review of the literature, case report and discussion of the role of retroperitoneal lymph node dissection. J Urol. (1970) 103(5):628–31. 10.1016/s0022-5347(17)62016-0 5443846

[39] Matei DV Rocco B Scardino E Spermatic cord liposarcoma: a report of four cases. Anticancer Res. (2003) 23(3C):2933–4. 12926138

[40] Matei DV Rocco B Scardino E Verweij F Renne G Fasani R Inguinal mass in a 66-year-old man. Urol Radiol. (1992) 14(1):62–4. 10.1007/BF02926904 1615577

[41] Schöffski P Chawla S Maki RG Italiano A Gelderblom H Choy E Eribulin versus dacarbazine in previously treated patients with advanced liposarcoma or leiomyosarcoma: a randomised, open-label, multicentre, phase 3 trial. Lancet. (2016) 387:1629–37. 10.1016/S0140-6736(15)01283-0 26874885

[42] Tawbi HA Burgess M Bolejack V Pembrolizumab in advanced soft-tissue sarcoma and bone sarcoma (SARC028): a multicentre, two-cohort, single-arm, open-label, phase 2 trial [published correction appears in Lancet Oncol. 2017 Dec;18(12):e711] [published correction appears in Lancet Oncol. 2018 Jan;19(1):e8]. Lancet Oncol. (2017) 18(11):1493–501. 10.1016/S1470-2045(17)30624-1 28988646 PMC7939029

[43] Tawbi HA Burgess M Bolejack V Van Tine BA Schuetze SM Hu J Results of a prospective phase 2 study of pazopanib in patients with advanced intermediate-grade or high-grade liposarcoma. Cancer. (2017) 123(23):4640–7. 10.1002/cncr.30926 28832986

[44] Samuels BL Chawla SP Somaiah N Staddon AP Skubitz KM Milhem MM Sustained clinico-radiologic response to anti-cytotoxic T lymphocyte antigen 4 antibody therapy in metastatic myxoid liposarcoma. J Oncol Pharm Pract. (2017) 23(7):549–51. 10.1177/1078155216664202 27511215

[45] Barone A Chi DC Theoret MR Chen H He K Kufrin D FDA Approval summary: trabectedin for unresectable or metastatic liposarcoma or leiomyosarcoma following an anthracycline-containing regimen. Clin Cancer Res. (2017) 23(24):7448–53. 10.1158/1078-0432.CCR-17-0898 28774898

[46] Bachmann R Rolinger J Girotti P Kopp HG Heissner K Amend B Liposarcoma of the spermatic cord: impact of final surgical intervention—an institutional experience. Int J Surg Oncol. (2016) 2016:4785394. 10.1155/2016/4785394 27190644 PMC4848420

